# Supplementary figures and images for: In vivo clonal analysis reveals spatiotemporal regulation of thalamic nucleogenesis
Source: PLoS Biol. 2018 Apr 23;16(4):e2005211. doi: 10.1371/journal.pbio.2005211 (PMC5933804; doi:10.1371/journal.pbio.2005211)

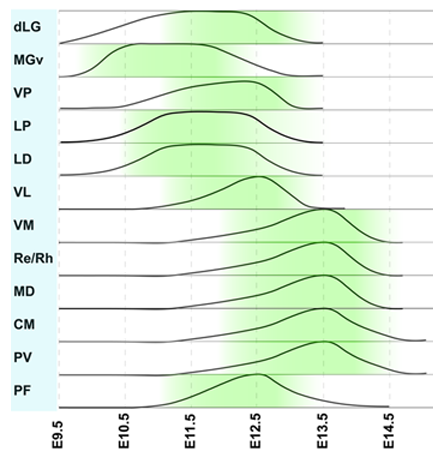

Supplement: S1 Fig — Embryonic stages at the bottom shows the stage of EdU administration. CM, centromedian; dLG, dorsal lateral geniculate; EdU, ethynyldeoxyuridine; LD, laterodorsal; LP, lateral posterior; MD, mediodorsal; MGv, ventral medial geniculate; PF, parafascicular; PV, paraventricular; Re/Rh, reuniens/rhomboid; VL, ventrolateral; VM, ventromedial; VP, ventral posterior. (TIF) [file pbio.2005211.s001.tif]

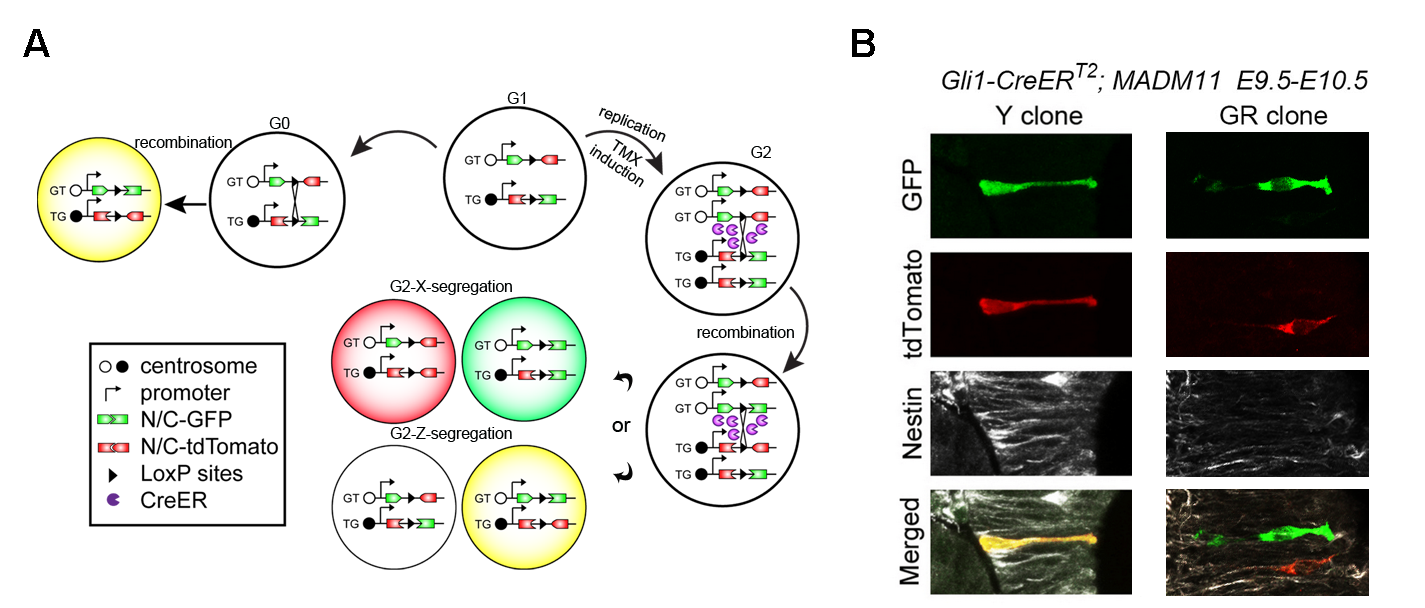

Supplement: S2 Fig — (A) In the MADM system, TMX-inducible Cre mice are used to drive interchromosomal recombination of the gene locus that contains partial coding sequences of EGFP and tdTomato (MADM-11GT and MADM-11TG). The recombination causes the production of fluorescent EGFP or tdTomato proteins. When the recombination occurs in a progenitor cell during G2 phase of the cell cycle, the 2 daughter cells express different combinations of fluorescent proteins depending on the mode of segregation of sister chromatids. Upon “G2-X”–type segregation, one daughter cell expresses EGFP (green or “G” cell), and the other expresses tdTomato (red or “R” cell). If these daughter cells undergo further divisions, respective fluorescent proteins continue to be expressed in the progeny, generating a “G/R clone” that contains a mixture of green and red cells. The other, “G2-Z”–type segregation produces one daughter cell that expresses both EGFP and tdTomato (yellow or “Y” cell), and the other daughter cell that expresses neither of the proteins. Thus, the G2-Z segregation generates a “Y clone,” which allows us to trace only half of the progeny of the recombined progenitor cell. (B) Immunostaining of an E10.5 section of Gli1CreERT2-labeled clones with anti-Nestin antibody. TMX was administered at E9.5. EGFP, enhanced green fluorescent protein; MADM, mosaic analysis with double markers; td, tandem dimer; TMX, tamoxifen. (TIF) [file pbio.2005211.s002.tif]

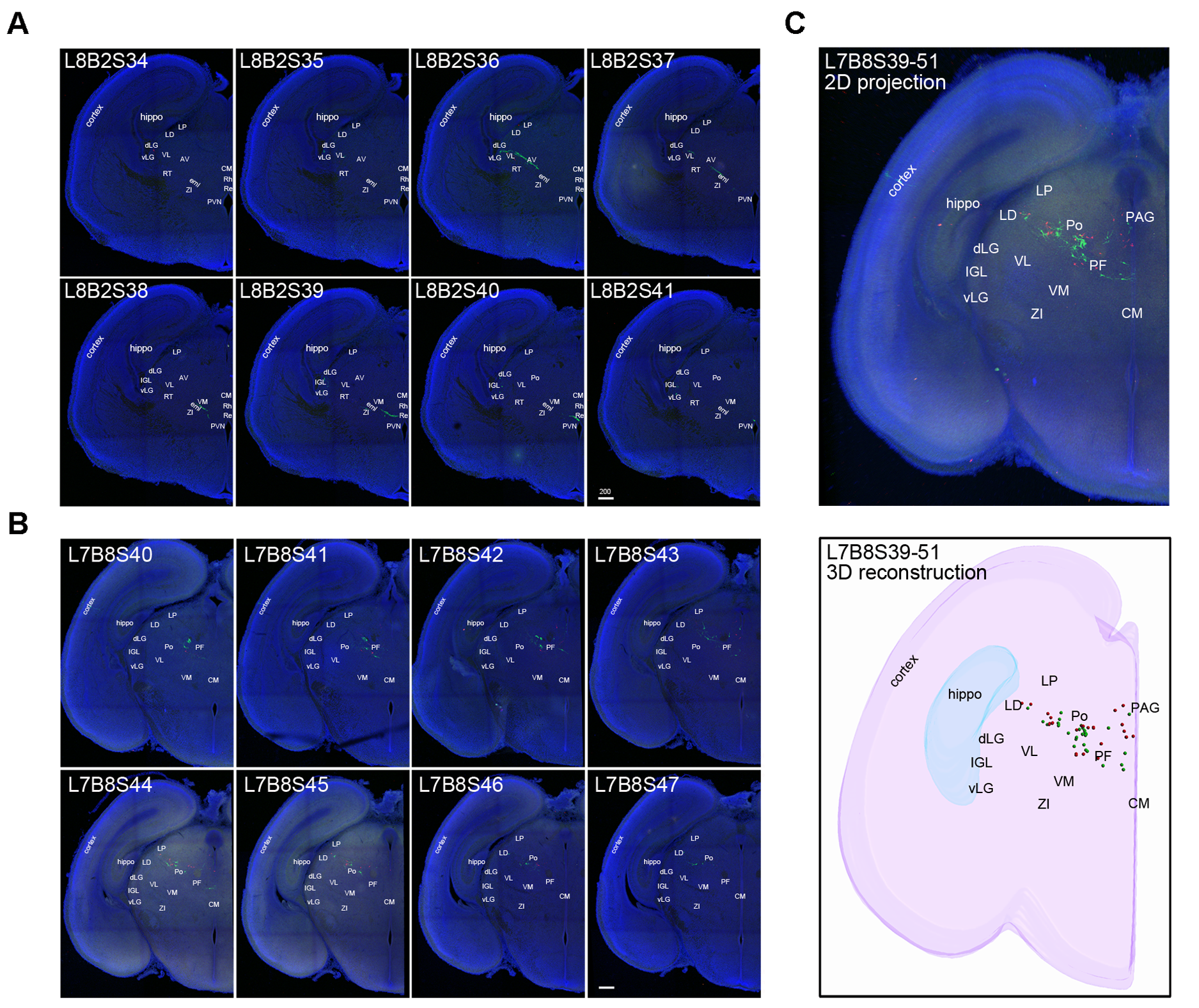

Supplement: S3 Fig — (A—B) Sample serial confocal images from 2 E18.5 Gli1 MADM clones (L8B2 and L7B8). Each image is a confocal Z-stack of an individual section (40-μm thick) and shows the distribution of green and red cells in multiple thalamic nuclei. L8B2S34 to L8B2S41 as well as L7B8S40 to L7B8S47 represent 8 consecutive sections. Scale bar: 200 μm. (C) 2D projection and 3D reconstruction of an entire E18.5 Gli1 MADM clone (L7B8) encompassing 13 sections. This clone lacks a retained RGC. MADM, mosaic analysis with double markers; RGC, radial glial cell. (TIF) [file pbio.2005211.s003.tif]

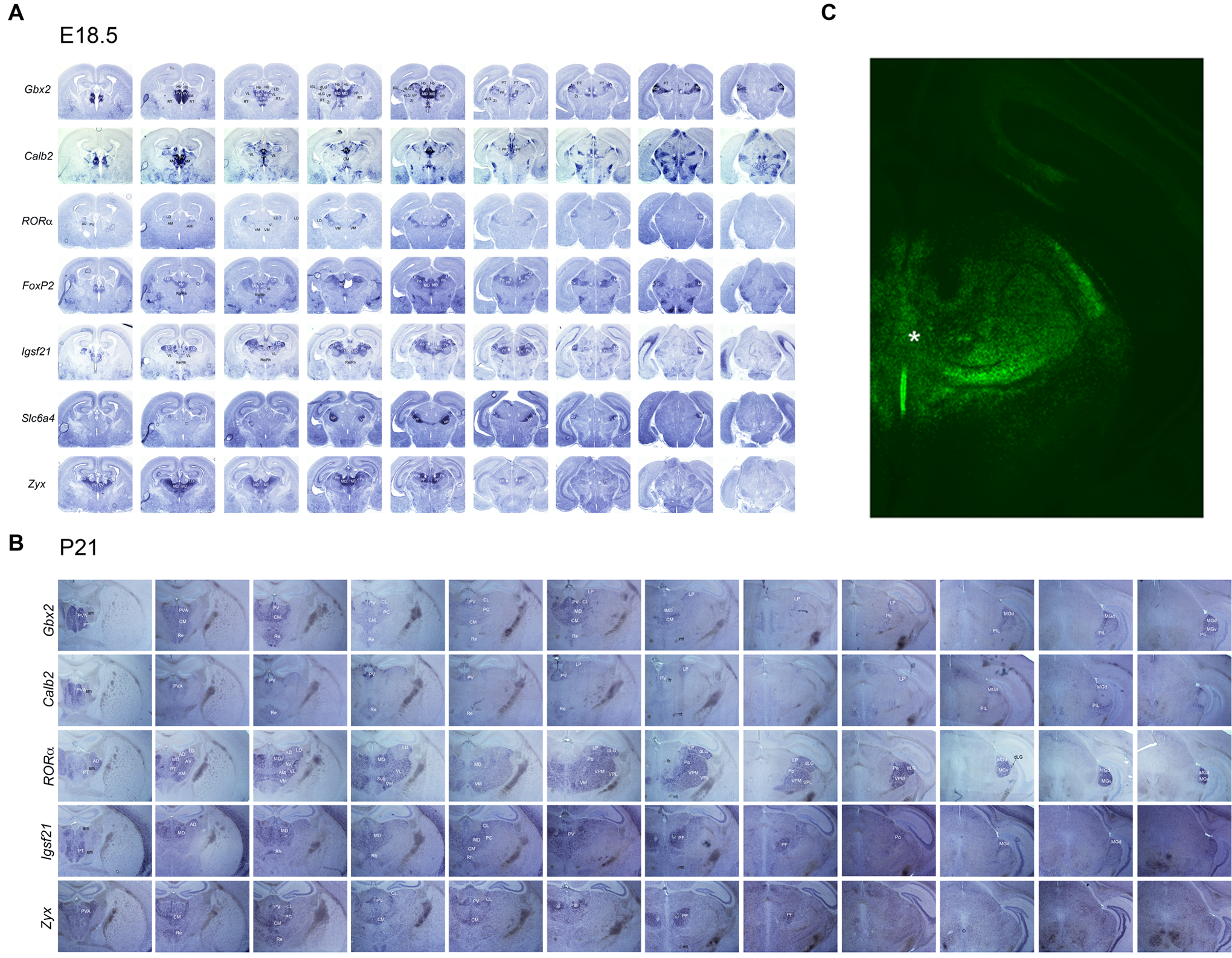

Supplement: S4 Fig — A custom atlas generated by in situ hybridization to define individual thalamic nuclei at (A) E18.5 and (B) P21. Expression of 7 representative markers is shown. This is a consecutive set of 40-μm-thick frontal sections. The left column is most dorsal, and the right column is the most ventral (see Fig 1A for axial orientation within the thalamus). Scale bar: 1 mm. (C) An image of frontal section from Olig3CreERT2;ZSGreen brain to show labeling of the medial ventral field (asterisk) by ZSGreen. Tamoxifen was administered at E9.5. (TIF) [file pbio.2005211.s004.tif]

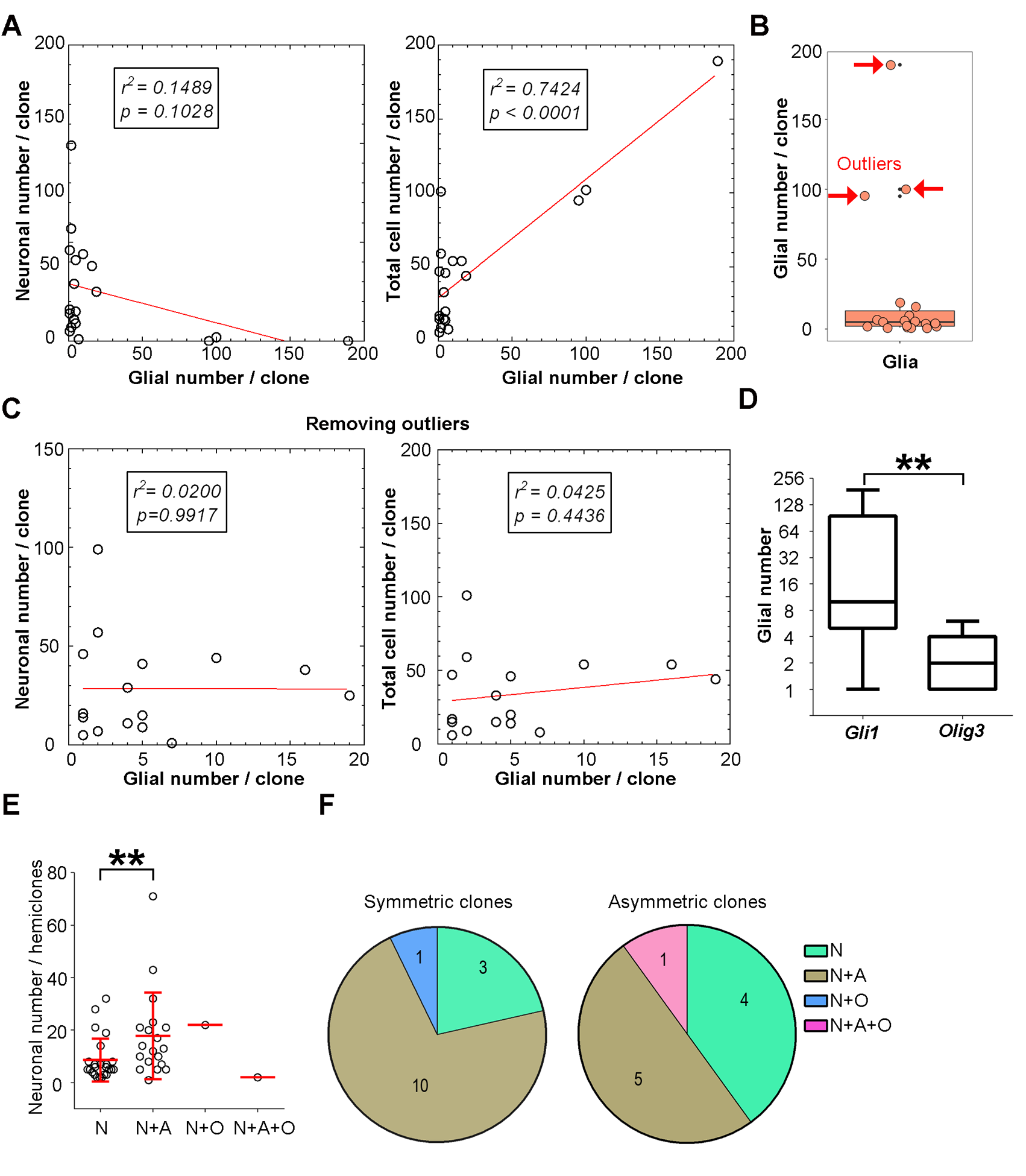

Supplement: S5 Fig — (A) Scatter plots illustrating the relationship between the number of glia and neurons (left) or total cell number (right) in the P21 clones derived from both Gli1 and Olig3 brains. While the correlation between glial and neuronal number is not striking, the glial number is linearly correlated with total cell number in the postnatal clones. r2, linear correlation coefficient; we refer to statistically significant as P < 0.05. (B) The box plot overlaid with dot plot showing the distribution of glial number per clone. The red arrows indicate the outlier clones beyond the Gaussian distribution. These clones consisted mostly of glial cells. (C) The linear correlation analysis shows that there is no significant correlation between glial and neuronal number (left) or glial and total cell number (right) after removing the outlier clones from P21 brains. (D) The box plot showing the glial cell number in the Gli1 and Olig3 clones from P21 brains. **P < 0.01 (Mann Whitney test). (E) Dot plot displaying the neuronal number in the hemiclones that contain N, N+A, N+O, or N+A+O. Each dot represents one hemiclone, and the red lines represent mean ± SEM. **P < 0.01 (Mann Whitney test). (F) Pie chart showing the percentage of symmetric proliferative and asymmetric neurogenic clones that contain N, N+A, N+O, or N+A+O. N, neurons only; N+A, neurons and astrocytes; N+O, neurons and oligodendrocytes; N+A+O, neurons, astrocytes, and oligodendrocytes. (TIF) [file pbio.2005211.s005.tif]

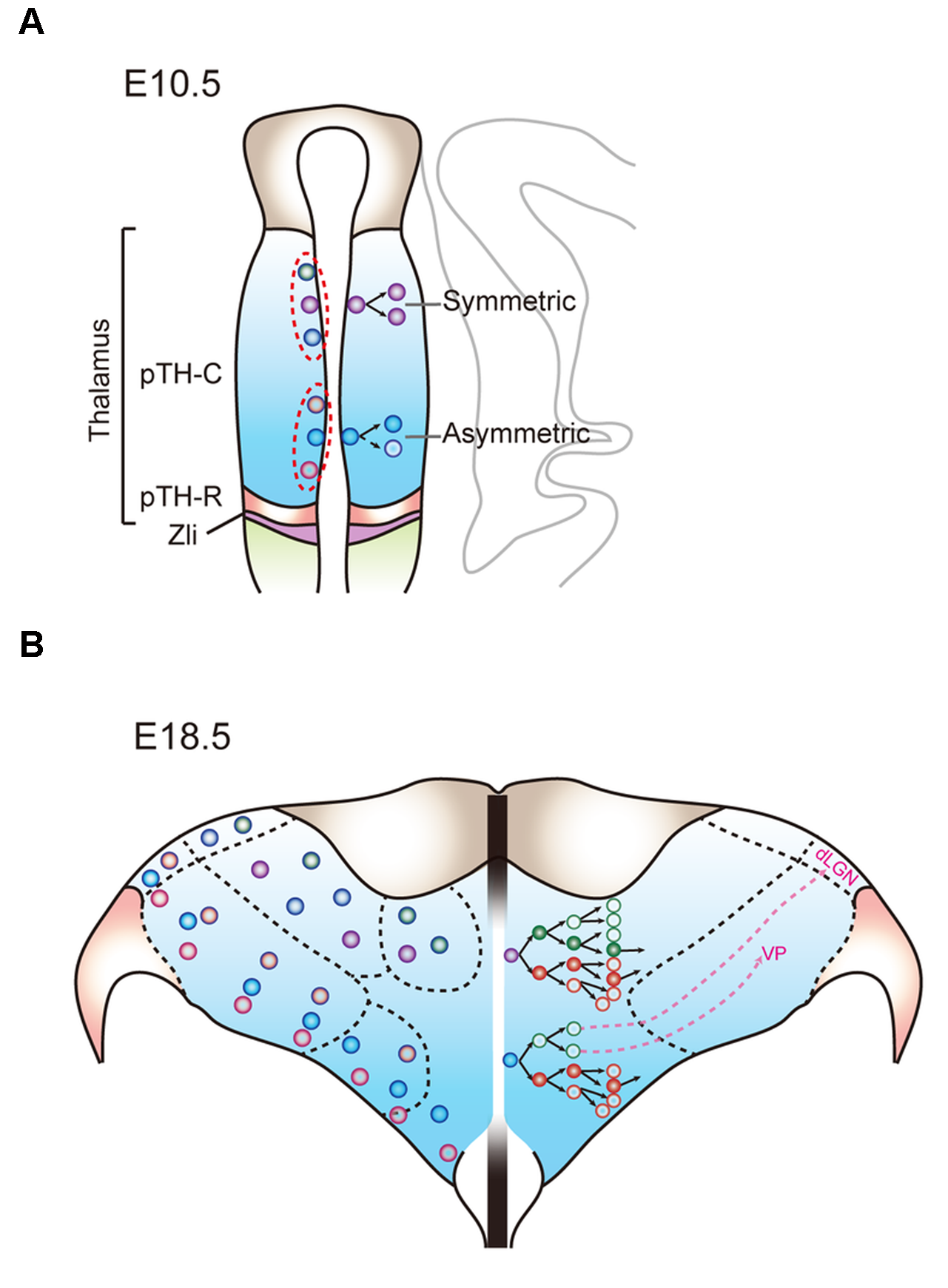

Supplement: S6 Fig — A schematic summary of the current study showing the main principles underlying spatiotemporal regulation of thalamic progenitor cell specification at (A) E10.5 and (B) E18.5. By E10.5, progenitor cells in the rostral-ventral part of the pTH-C domain are already undergoing asymmetric divisions (panel A; dots in the lower part of the pTH-C domain on the right side; see also Fig 1A) and produce neurons that later populate principal sensory nuclei including VP and dLG. (B) The long-term lineage tracing shows that, within the cell lineages that are derived from rostral-ventral progenitor cells, earlier-born neurons populate laterally located, principal sensory nuclei including dLG and VP, whereas later-born neurons populate more medial nuclei (dots in the lower part of the thalamus in panel B). In contrast, progenitor cells at more caudo-dorsal locations are still mainly undergoing symmetric division at E10.5 (panel A; dots in the upper part of the pTH-C domain on the right side) and eventually produce neurons in caudo-dorsally located nuclei (dots in the upper part of the thalamus in panel B). Regardless of cell positioning, a majority of radial glial precursors undergo either symmetric proliferative or asymmetric neurogenic division in the first round of cell division after genetic labeling at E10.5 (see also Fig 3D). On the left side of schematics, (A) progenitor cells and (B) their progeny are color coded to indicate their lineage relationship. dLG, dorsal lateral geniculate; pTH-C, caudal thalamic progenitor domain; VP, ventral posterior. (TIF) [file pbio.2005211.s006.tif]
